# A Framework for Corticomuscle Control Studies Using a Serious Gaming Approach

**DOI:** 10.3390/mps8040074

**Published:** 2025-07-07

**Authors:** Pedro Correia, Carla Quintão, Cláudia Quaresma, Ricardo Vigário

**Affiliations:** 1 Physics Department, NOVA School of Science and Technology, NOVA University of Lisbon, 2829-516 Caparica, Portugal; pml.correia@campus.fct.unl.pt (P.C.); cmquintao@fct.unl.pt (C.Q.); q.claudia@fct.unl.pt (C.Q.); 2 Laboratory of Instrumentation, Biomedical Engineering and Radiation Physics (LIBPhys-UNL), NOVA School of Science and Technology, NOVA University of Lisbon, 2829-516 Caparica, Portugal; 3 Associated Laboratory in Translation and Innovation Towards Global Health (REAL), 2829-516 Caparica, Portugal

**Keywords:** EEG, EMG, cortico-muscle communication, corticomuscular control, serious gaming, phase synchrony, reference phase analysis

## Abstract

Sophisticated voluntary movements are essential for everyday functioning, making the study of how the brain controls muscle activity a central challenge in neuroscience. Investigating corticomuscular control through non-invasive electrophysiological recordings is particularly complex due to the intricate nature of neuronal signals. To address this challenge, we present a novel experimental methodology designed to study corticomuscular control using electroencephalography (EEG) and electromyography (EMG). Our approach integrates a serious gaming biofeedback system with a specialized experimental protocol for simultaneous EEG-EMG data acquisition, optimized for corticomuscular studies. This work introduces, for the first time, a method for assessing brain–muscle functional connectivity during the execution of a demanding motor task. By identifying neuronal sources linked to muscular activity, this methodology has the potential to advance our understanding of motor control mechanisms. These insights could contribute to improving clinical practices and fostering the development of novel brain–computer interface technologies.

## 1. Introduction

In the human body, multiple organ systems interact dynamically to sustain health and function. Mapping these networks is crucial for understanding fundamental physiological processes and for distinguishing between physiological and pathological behaviors [1,2].

A particularly important area of research is the complex interaction between the brain’s cortex and muscles, known as corticomuscular control, which underlies human movement. Advancing our understanding of this interaction could facilitate the development of novel technologies, such as brain–computer interfaces, and improve clinical rehabilitation strategies.

In this context, research on corticomuscular control holds significant potential for enhancing rehabilitation programs for individuals with motor impairments [3,4]. Specifically, understanding the physiological mechanisms affected in different clinical conditions is essential not only for designing targeted rehabilitation interventions but also for assessing prognosis and monitoring treatment outcomes.

Recently, corticomuscular interactions have attracted increasing attention within the neuroscience community, leading to the proposal of several mechanisms of communication between cortical and muscular activity. One widely accepted metric of functional connectivity is corticomuscular coherence (CMC), which quantifies the reciprocal interplay between neuronal and muscular assemblies [4].

An increasing number of studies have explored the role of neural synchronization between cortical and muscular systems in motor control, particularly focusing on CMC and its modulation across different motor tasks and physiological conditions. Research has demonstrated that motor tasks are often accompanied by increased EEG-EMG coherence, particularly in the beta frequency band (13–30 Hz), among healthy individuals [5]. This coherence reflects the functional coupling between cortical motor areas and the corresponding muscles, providing valuable insights into the neural mechanisms underlying voluntary movement [6].

Early evidence from Conway et al. [7] demonstrated significant beta-band coherence (13–35 Hz) between cortical activity and EMG during isometric contractions, linking cortical rhythms to motor unit synchronization. Subsequent work by Feige et al. [8,9] and Kilner et al. [9] showed that CMC is not static but dynamically modulated by task features such as movement phase, frequency, and mechanical load. Toma et al. [10] observed that faster repetitive movements (3–4 Hz) induce sustained cortical coupling, whereas slower movements result in transient activation and deactivation cycles. Coherence patterns also shift during movement planning and execution, as highlighted by Wheaton et al. [11], who identified preparatory beta-band synchronization between parietal and premotor regions during hand gesture tasks. Additionally, Perez et al. [12] demonstrated increased beta-range coherence during high-force bilateral tasks. These findings suggest a role for oscillatory coupling in motor coordination and control, evidencing that the degree of CMC reflects the level of control demand imposed by a given motor task, with more complex or demanding tasks eliciting stronger or more sustained cortical–muscular coupling.

Clinical studies revealed atypical CMC in neurodegenerative and post-stroke populations. For instance, Bista et al. [13] found altered CMC patterns in patients with primary lateral sclerosis, while Parmar et al. [5] reported compensatory beta-band synchronization in stroke survivors, indicating adaptive or maladaptive plasticity in motor networks. Furthermore, disruptions in normal corticomuscular coherence have been associated with various motor impairments, including stroke [14,15,16], Parkinson’s disease [17,18], essential tremor [19], multifocal high-frequency rhythmic myoclonus [20], and cerebral palsy [21].

While the aforementioned studies assessed CMC directly at the sensor level between the EEG/MEG and EMG signals, other studies have employed source localization methods to improve the spatial specificity of coherence estimates. For example, Kilner et al. [22] demonstrated the task-dependent modulation of EMG-EMG coherence and localized the cortical sources of MEG-EMG coherence during sustained grip using a single equivalent current dipole model, identifying the hand region of the contralateral motor cortex as a likely origin. Similarly, Gross et al. [23] applied spatial filtering to MEG signals and found significant beta-band coherence (∼20 Hz) between the estimated primary motor cortex (M1) sources and EMG during isometric contraction. They further showed that phase delays between cortical and muscular activity matched corticospinal conduction times, reinforcing the notion of the cortical driving of spinal motoneurons.

In another study, Gross et al. [24] used Dynamic Imaging of Coherent Sources (DICS) to localize M1 and supplementary motor area (SMA) sources during unimanual and bimanual tasks. They observed the task-specific modulation of beta- and gamma-band coherence between the cortex and muscles and across cortical regions, with gamma-band coherence peaking during isometric contractions and beta-band coherence varying between dynamic and static conditions. This supports a frequency-dependent functional organization within the motor system.

Schoffelen et al. [25] also utilized spatial filtering techniques and a two-dipole source model to estimate the corticomuscular coherence patterns. They found spatially localized beta-band coherence in the contralateral motor cortex, as well as coherent clusters in the ipsilateral motor cortex and cerebellum, suggesting that motor control engages a distributed network beyond M1.

These findings underscore the relevance of CMC as a reliable biomarker of motor control and corticomuscular connectivity across both healthy and clinical populations.

Beyond coherence, growing evidence suggests that phase synchronization plays a fundamental role in brain-to-brain [25,26,27,28,29,30,31,32,33] and brain-to-muscle interactions [23,34]. Unlike coherence, phase synchronization is independent of signal amplitude, making it a more robust and reliable measure of corticomuscular coupling [35,36]. Additionally, phase relations provide deeper insights into the temporal dynamics of neuronal activity. Notably, disturbances in healthy brain synchronization patterns have been associated with various neurological disorders, including Alzheimer’s disease, Parkinson’s disease, and autism [28,30,37,38].

While traditional CMC and phase synchronization analyses have provided valuable insights, several methodological limitations constrain their reliability and interpretability. A fundamental concern is that coherence metrics are inherently influenced by the amplitude of the underlying signals [39,40]. As a result, fluctuations in signal magnitude that are not functionally related to actual corticomuscular coupling can artificially inflate coherence estimates and lead to potentially misleading interpretations of connectivity strength. Furthermore, many conventional approaches assess coherence or phase directly at the sensor level without applying neuronal source separation techniques [41]. This lack of spatial specificity increases the risk of spurious synchronization due to volume conduction or the mixing of neural and non-neural sources [42,43], thereby compromising the physiological validity of the observed interactions.

Although corticomuscular interactions have been extensively studied, the underlying neural mechanisms remain insufficiently understood, highlighting the need for more advanced analytical and methodological approaches. While sophisticated signal processing techniques are essential, the design of refined experimental paradigms plays an equally crucial role. Effective corticomuscular research protocols should involve motor tasks that require precise control and sustained attention, while minimizing potential confounding factors, such as excessive cognitive demands or distracting external stimuli.

Developing an optimized experimental methodology for studying corticomuscular control is therefore of paramount importance. A well-structured approach enhances research specificity by controlling key variables, ultimately contributing to advancements in neuroscience. To address the current lack of protocols specifically tailored to study corticomuscular control, this study protocol proposes an innovative approach that combines serious gaming with EEG-EMG recordings to investigate the neural control of fine pinch grip movements. The methodology will be applied to investigate corticomuscular control through phase synchronies in children with cerebral palsy.

In contrast to conventional CMC analysis, phase synchrony provides greater precision and reliability in detecting transient coupling among oscillatory neural signals, making it especially effective for capturing dynamic corticomuscular connectivity. The proposed study protocol introduces Reference Phase Analysis (RPA), an advanced analytical pipeline encompassing neuronal source separation based on phase synchrony between cortical activity and EMG. This methodology directly addresses the key limitations of traditional CMC approaches—mitigating signal amplitude dependency by utilizing phase-coupling metrics instead of coherence and minimizing spurious synchronization caused by volume conduction or signal mixing through the application of synchronous source separation—thus enabling a more accurate and physiologically grounded assessment of corticomuscular coupling.

To better understand the physiological and pathological mechanisms underlying motor control, this study aims to investigate potential differences in phase synchronization between children with cerebral palsy and healthy individuals. Specifically, it seeks to answer the following research question: To what extent does motor control, assessed through phase synchrony, differ between children with cerebral palsy and typically developing children?

The primary objective of this study protocol is to assess motor control in children with cerebral palsy by analysing phase synchrony between EEG and EMG signals. This methodology aims to explore corticomuscular connectivity patterns that underlie motor coordination deficits in this population, providing insights into the neural mechanisms associated with impaired motor function.

Additionally, this study aims to investigate physiological cortical activation patterns during the execution of precise and fine hand motor tasks in healthy individuals. It further seeks to identify and characterize neuronal sources synchronized with muscular activity, with a particular focus on their spatial localization and the magnitude of synchrony through PLV analysis. Finally, comparing these cortical activation patterns and their corresponding neuronal sources with muscular activity in children with cerebral palsy against typical physiological patterns observed in healthy individuals is expected to provide critical insights into the neural discrepancies associated with motor impairments.

## 2. Materials and Methods

This study will be conducted according to the guidelines of the Declaration of Helsinki. Ethical approval was received from the NOVA School of Science and Technology Ethics Committee (CE_FCT_006-2023), and written informed consent will be provided by all participants.

This section details the materials and equipment used in this study (Section 2.1), including the force actuator, EEG-EMG system, consumables, and supporting technology required for the experiments. Section 2.2 and Section 2.3 describe the characteristics, inclusion, and exclusion criteria for the control and experimental groups, respectively. Additionally, Section 2.4 introduces the biofeedback system designed to facilitate consistent performance during the motor control task. Section 2.5 presents a comprehensive overview of how electrophysiological data will be recorded and how the experimental protocol was defined. Finally, Section 2.6 outlines the data processing methods that will be applied to the collected EEG and EMG signals, covering preprocessing, synchronous source separation, phase-locking analysis, and brain source localization to investigate corticomuscular coupling.

### 2.1. Materials

#### 2.1.1. Force Actuator

- Binder Paper Clip;
- 360 Ω and 120 Ω Resistors;
- Dotted Printed Circuit Board (PCB);
- Arduino ATmega328;
- Instrumentation amplifier AD620;
- BF350 strain gauge sensors.

#### 2.1.2. EEG-EMG System, Software, and Accessories

- g.Nautilus 32 Multi-Purpose g.SCARABEO (28 + 4) device;
- g.tec Suite 2020;
- g.Recorder Basic;
- g.GAMMAcap g.Nautilus, 2 mm;
- g.SCARABEO syringe;
- 3 clip-leads, 150 cm, 1.5 mm safety connector, for disposable electrodes;
- Chronometer;
- MATLAB software (version R2025a) [44];
- Processing software [45].

#### 2.1.3. EEG-EMG Consumables

- Electrolyte gel compatible with the EEG system (e.g., Parker signagel);
- Cleaning tools for EEG electrodes;
- Disposable Electrodes (e.g., Kendall™ H124SG Electrodes, CardinalHealth™).

#### 2.1.4. Supporting Technology Requirements

- Computer running a Microsoft Windows operating system (Windows 10 Professional English with 64 Bit);
- LED Monitor.

### 2.2. Control Group

Participants in the control group will be selected according to the following criteria: a minimum sample size of 30 (N ≥ 30), an age range between 8 and 14 years, and a balanced gender distribution, with 50% female (F) and 50% male (M) participants. Individuals will be excluded if they are diagnosed with neurological or neuromuscular disorders, are taking medications that affect the central nervous system, or have scalp lesions.

### 2.3. Experimental Group

Participants in the experimental group will meet the following inclusion criteria: a minimum sample size of 30 (N ≥ 30), an age range between 8 and 14 years, a clinical diagnosis of hemiplegic or diplegic cerebral palsy, and an equal gender distribution of 50% female (F) and 50% male (M) participants. Exclusion criteria will include muscular impairment in both upper limbs, cognitive impairment, and the presence of scalp lesions.

### 2.4. Biofeedback System for Motor Control Task

**Figure 1:**
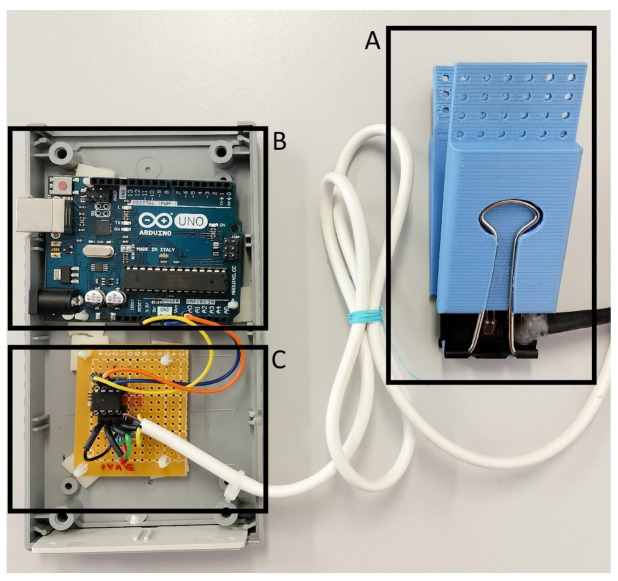
Force actuator used in the Gripping Control Game. (**A**)—Binder paper clip with two BF350 strain gauge sensors embedded on its inner and outer surfaces. (**B**)—Arduino UNO (ATmega328) microcontroller. (**C**)—Electronic circuit including a Wheatstone bridge and an AD620 instrumentation amplifier.

A biofeedback system was developed to ensure the reproducibility of the gripping control task across trials and participants. This system consists of a force actuator (Figure 1) and a gaming computer interface, referred to as the “Gripping Control Game”.

**Figure 2:**
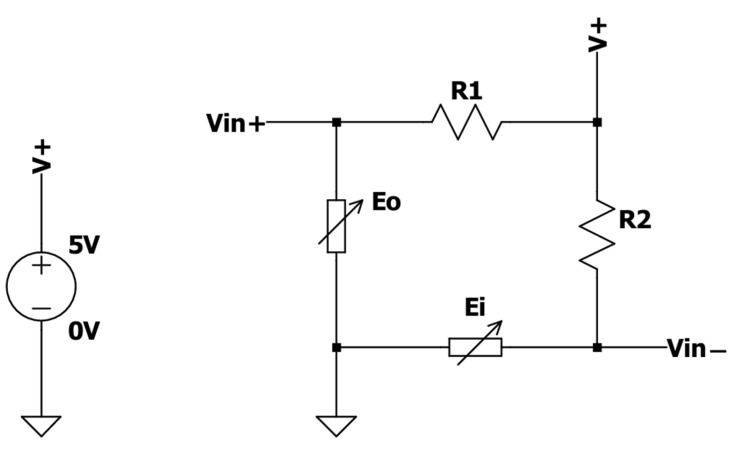
Schematic of the Wheatstone bridge, composed of two BF350 strain gauge sensors (*E_i_* and *E_o_*), attached to a binder clip and two 360 Ω resistors (R1 and R2), where *Vin*^+^ and *Vin*^−^ correspond to the non-inverting and inverting input of the AD620 amplifier.

To measure the force applied to a binder paper clip, two BF350 strain gauge sensors were embedded on its inner and outer surfaces. The resistance of these sensors varies as a function of the applied strain; the resistance increases when the strain gauge is stretched and decreases when it is compressed. These electronic components were integrated into a Wheatstone bridge circuit, along with two 360 Ω resistors, as illustrated in Figure 2. Since the voltage between *Vin*^+^ and *Vin*^−^ is given by Equation (1),

(1) $${Vin}^{+}-{Vin}^{-}=V^{+}\left(\frac{E_{i}}{E_{i}+R1}-\frac{E_{o}}{E_{o}+R2}\right)$$

the Wheatstone bridge was designed to detect tiny resistance changes in both strain gauges. As the variations in $E_{i}$ and $E_{o}$ are symmetrical (when the inner surface of the binder clip is stretched, the outer is compressed), this circuit also improves the sensitivity by amplifying the effect of the resistance change. Consequently, the measurable potential difference at the bridge terminals serves as a function of the force applied to the actuator.

**Figure 3:**
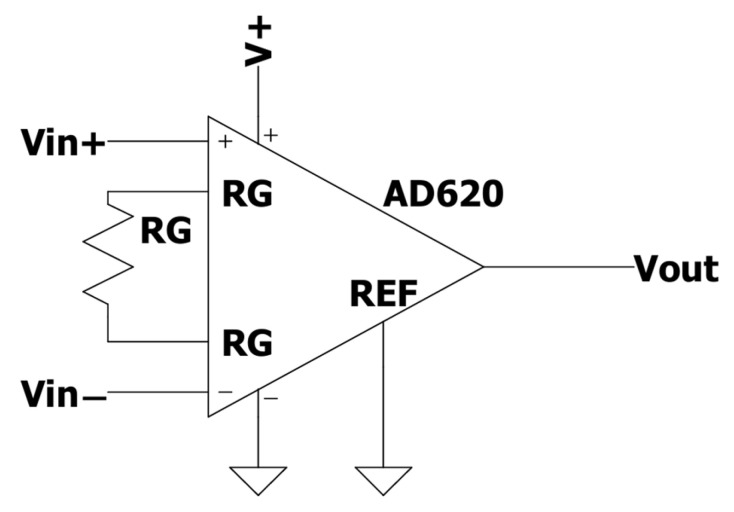
Schematic of the AD620 amplifier. Vout is the output to be converted to a digital signal.

To maximize the resolution of the actuator, the voltage difference between *Vin*^+^ and *Vin*^−^ was amplified by a factor of 413 using an AD620 instrumentation amplifier (Figure 3) with a resistance RG of 120 Ω. The electronic circuit was assembled on a perforated printed circuit board (PCB). Finally, the output signal was converted into a digital format and transmitted to a computer via USB, utilizing an Arduino UNO ATmega328 microcontroller.

**Figure 4:**
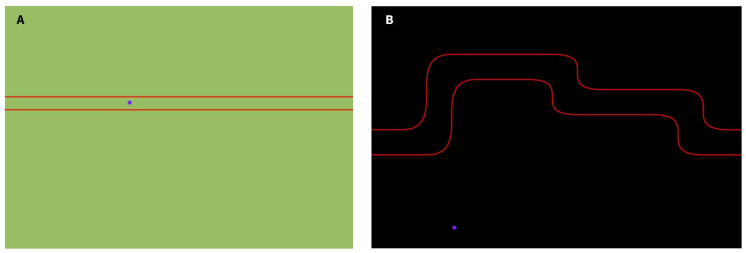
Gripping Control Game. (**A**,**B**) represent two different mazes, with different levels of difficulty designed for our experiment. A green background indicates that the cursor, represented by a purple circle, is positioned within the red path, whereas a black background implies that the cursor is outside of the intended path.

The Gripping Control Game, developed using Processing, presents participants with a maze path they must navigate during the motor task. The cursor’s vertical position over time is controlled by the force applied to the binder paper clip. When the cursor (displayed as a purple circle) remains within the designated path, marked by red boundary lines, the background turns green (Figure 4A), indicating good performance. If the cursor deviates from the intended path, the background turns black (Figure 4B), providing immediate feedback to the participant.

Several studies have demonstrated that synchrony is a transient phenomenon [38,46,47]. Additionally, preliminary tests conducted during the development of the RPA algorithm suggested that transient synchronization events related to corticomuscular control primarily occur during periods of force variation, when motor control demands are highest. This assumption guided the design of the serious game’s maze paths (Figure 4), which incorporate varying difficulty levels and require different force outputs to effectively study corticomuscular control.

### 2.5. Procedure

#### 2.5.1. Data Acquisition

The multi-purpose version of the g.Nautilus RESEARCH system allows for the simultaneous recording of EEG alongside other electrophysiological signals, such as ECG, EMG, and EOG. In the proposed experimental setup, both EEG and EMG channels will be configured for data acquisition.

When connecting EMG electrodes to the device, it is essential to bridge the ground (GND) and reference (REF) terminals using a jumper cable to minimize electrical noise. Consequently, while a GND electrode remains necessary for EEG recordings, an EEG channel must be repurposed as the new reference and positioned on the earlobe. In our experimental protocol, channel 16 will be designated as the reference, while EMG electrodes will be connected to channels 31 and 32.

**Figure 5:**
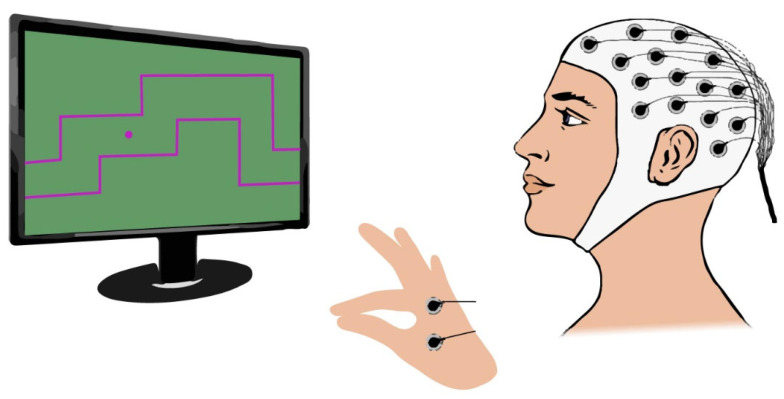
Experimental setup.

Since the occipital cortex is not expected to contribute significantly to corticomuscular control, three occipital EEG channels (PO7, Oz, and PO8) will be repurposed—one as the new EEG reference and the other two for EMG recordings. This modification will enable the synchronous acquisition of both the EEG and EMG signals. Figure 5 illustrates the proposed experimental setup, where channels 31 and 32 will be used as EMG channels.

**Figure 6:**
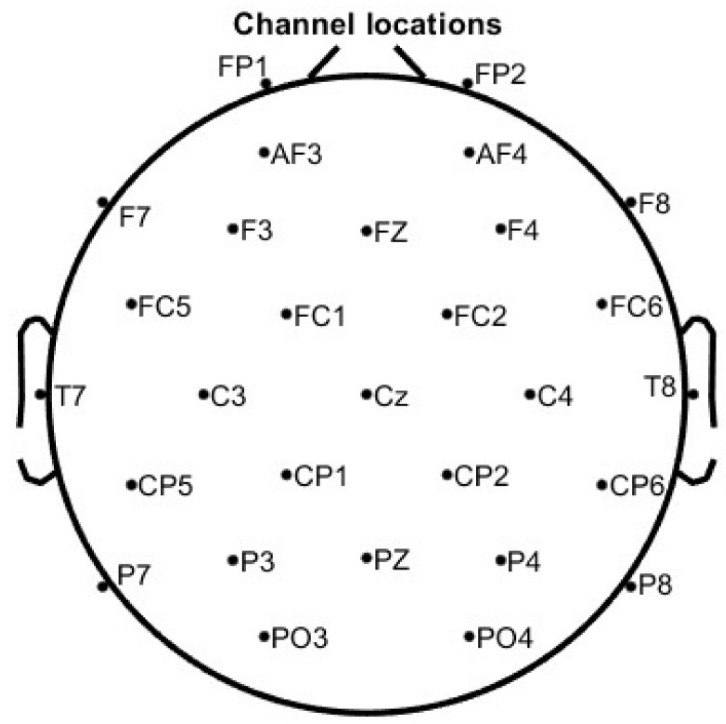
EEG electrode placement following an extended version of the International 10–20 system.

The placement of the 29 EEG electrodes will follow the extended version of the International 10–20 system, as shown in Figure 6. All EEG electrodes will be filled with conductive gel using a g.SCARABEO syringe, and electrode impedance will be maintained below 50 kΩ, in accordance with the manufacturer’s recommendations.

**Figure 7:**
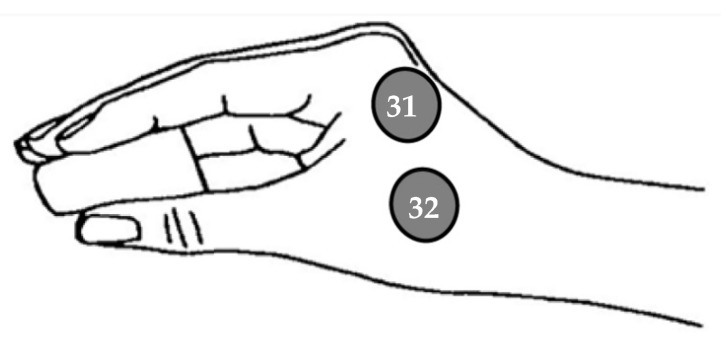
EMG electrode placement.

Additionally, two EMG electrodes will be placed as illustrated in Figure 7. This electrode placement was chosen to capture the muscular activity originating from the adductor pollicis and the first dorsal interosseus, both of which play a key role in the pinch grip movement [48,49,50].

EEG and EMG signals will be recorded using the g.Recorder software. All EEG channels will be configured in a bipolar arrangement relative to the new EEG reference (channel 16), while the EMG signals will be recorded in a bipolar configuration between channels 31 and 32. Electrophysiological data will be acquired at a sampling rate of 500 Hz, with an analog bandpass filter (0.01–60 Hz) and a notch filter at 50 Hz; both filters will be configured in the g.Recorder Basic system.

#### 2.5.2. Experimental Protocol

**Figure 8:**
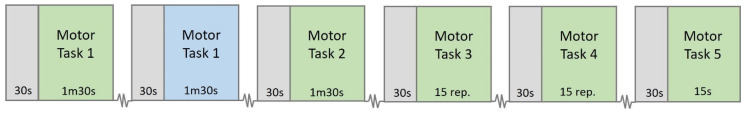
Timeline of the experimental protocol. Grey blocks represent relaxation moments between tasks, green blocks indicate motor tasks during which EMG is recorded from the dominant hand, and the blue block depicts a motor task where EMG is recorded from the non-dominant hand.

After pairing the g.Nautilus system with the base station and activating the electrodes with conductive gel, the device settings will be configured as illustrated in Figure 8. Subsequently, EEG and EMG data will be recorded simultaneously while participants perform the experimental protocol, which consists of five distinct motor tasks, all involving the pinch grip movement using the binder clip.

The first and second motor tasks will be guided by the Gripping Control Game presented in Section 2.3. In these tasks, participants will be asked to track the game cursor along a designated path displayed on the computer screen (paths A and B in Figure 3, respectively). As explained earlier, the cursor’s vertical position over time is controlled by the force applied to the binder paper clip: when the clip is tightened, the cursor moves up; when it is loosened, the cursor moves down. Tasks 1 and 2 will last 90 s each.

In the first task, participants will use both hands, one for each row, while EMG data is acquired from the corresponding hand. In the subsequent tasks, EMG signals will be collected only from the participant’s dominant hand.

In Task 3, the participant will use one hand to repeatedly squeeze and release a paper binder clip. Participants will perform a total of 15 contraction–relaxation cycles at a steady pace of one cycle every 2 s (0.5 Hz). During each contraction phase, the participant should squeeze the clip fully, opening it to its maximum extent. A researcher will provide verbal cues such as “squeeze” and “release” to guide the participant in maintaining the correct pace and completing the full range of motion for each cycle.

In the fourth task, participants will hold a paper binder clip in each hand and perform the same squeeze-and-release movement as in Task 3. This time, the movement will be done with both hands simultaneously; however, EMG signals will be collected only from the participant’s dominant hand. Participants will complete 15 contraction–relaxation cycles, maintaining the same pace of one cycle every 2 s (0.5 Hz), aiming to open each clip fully during the squeeze phase.

For the final motor task, participants will be asked to squeeze the binder clip with their maximum force and hold the contraction for 15 s using one hand. This task is designed to assess sustained maximal grip strength.

The inclusion of diverse motor tasks in this protocol was intentionally designed to explore different aspects of motor control by examining phase synchrony between the EEG and EMG signals. Since phase synchrony is expected to be transient and task-dependent, the protocol incorporates tasks involving steady force, as well as abrupt and gradual force changes.

Each of these tasks is associated with distinct motor control strategies. For example, maintaining a steady grip requires continuous, stable motor output, while sudden or smoothly shifting force levels challenge the participant’s ability to modulate force quickly and accurately. By incorporating this range of motor actions, the protocol enables a more nuanced investigation of how phase synchrony adapts in response to varying biomechanical and neural control requirements.

To standardize task execution and ensure that all participants perform the tasks in a consistent and replicable manner, the study utilizes a serious gaming interface in Tasks 1 and 2. This system provides real-time visual feedback, guiding participants to exert the correct force output. It also serves as a means of ensuring uniform task presentation and execution across individuals, which is crucial for minimizing inter-subject variability and increasing the reliability of the data.

Additionally, Task 1 was also performed with the non-dominant hand. This allows the study to explore potential asymmetries in motor control and the lateralization of neural processing between the dominant and non-dominant hands. Such comparisons can shed light on how motor control mechanisms may differ depending on limb dominance.

To account for the potential influence of cognitive and visual factors on phase synchrony, certain tasks were deliberately performed without the gaming interface. By removing visual feedback and interactive elements, these conditions were designed to minimize the impact of attention, visual processing, and decision-making demands. This approach allows for a more focused analysis of motor-related neural activity, helping to determine whether the observed EEG-EMG phase synchrony is primarily driven by motor control processes or modulated by broader cognitive influences.

Finally, bilateral tasks were included to assess whether using both hands simultaneously engages motor networks differently compared to unilateral movements. Overall, the protocol was designed to be as comprehensive as possible, enabling the study of motor control across a range of force levels and coordination demands.

The proposed experimental design is depicted in Figure 8.

### 2.6. Data Processing

This protocol describes the data processing steps designed to investigate corticomuscular coupling during fine motor control tasks in healthy individuals. The approach involves preprocessing EEG and EMG signals, identifying synchronized neuronal sources, and localizing brain regions consistently coupled with muscular activity. The main processing steps are as follows:

- Preprocessing:
  EEG and EMG will be bandpass filtered (13–30 Hz), downsampled to 60 Hz, and artifacts will be removed from the EEG signals using ICA.
- Temporal Decorrelation Source Separation (TDSEP):
  TDSEP will be employed to separate EEG sources while preserving the physiological temporal structure. Coherence will be evaluated between the EMG signal and TDSEP sources. This step allows for the identification of the frequency bands and time intervals where maximum coherence occurs, which will be used in subsequent analysis.
- RPA:
  RPA will identify EEG sources phase-locked with EMG by maximizing the PLV between them using an adaptive gradient ascent algorithm.
- Localization:
  The FieldTrip toolbox will be used to localize brain sources, showing consistent synchrony with muscular activity.

In the rest of this section, each processing step mentioned before will be described and discussed in detail.

In this study, a bandpass filter between 13 and 30 Hz will be applied to the EEG and EMG signals to focus the analysis on the beta frequency band [51]. The beta band was chosen because beta-band activity recorded at the sensorimotor areas has been shown to play a functional role in the execution of motor actions [5].

The beta band is strongly associated with sensorimotor processes and is known to play a key role in motor control and corticomuscular coupling. Previous research has demonstrated that oscillatory activity in the beta range reflects important aspects of movement preparation, execution, and fine motor coordination [6]. Restricting the analysis to the 13–30 Hz frequency range isolates the neural oscillations relevant to motor control while minimizing interference from unrelated lower-frequency (e.g., alpha) and higher-frequency (e.g., gamma) activity. This targeted filtering step ensures that subsequent analyses are more sensitive and specific to motor control.

After bandpass filtering the signals between 13 and 30 Hz, the data will be downsampled to 60 Hz to optimize the computational efficiency without compromising the integrity of the information of interest. Since the maximum frequency content after filtering is 30 Hz, according to the Nyquist theorem, a sampling rate of at least 60 Hz is sufficient to accurately represent the signals without aliasing.

Additionally, artifact components that could compromise the quality and reliability of subsequent analyses will be removed from the EEG signals using Independent Component Analysis (ICA). These components are identified through the visual inspection of both the independent component time courses and their corresponding topographic maps, allowing for the recognition of typical artifact patterns such as eye blinks, muscle activity, or impedance artifacts [52]. Once identified, the artifact-related components are excluded, and the clean EEG signal is reconstructed. This process ensures that the analysis focuses on meaningful neuronal activity, thereby improving the signal-to-noise ratio and enhancing the accuracy of coherence and phase-locking measures.

**Figure 9:**
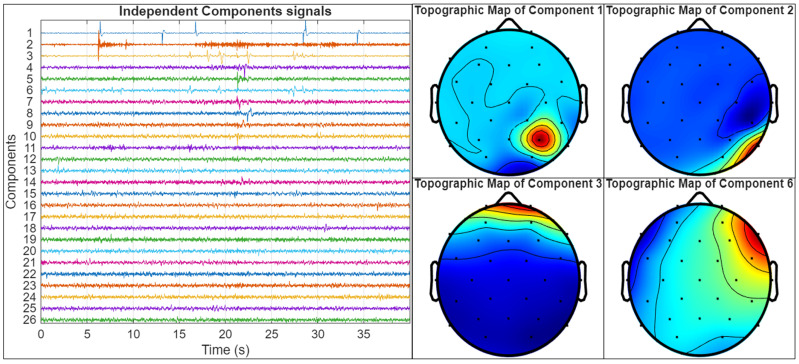
Example of artifact components identified using Independent Component Analysis (ICA). On the left, the time courses of the 26 independent components are shown. On the right, the topographic maps of four selected components (1, 2, 3, and 6) are displayed. Component 1 corresponds to an impedance artifact, Component 2 to EMG (muscle) activity, and Components 3 and 6 to ocular artifacts. These components were identified and removed through visual inspection based on their characteristic temporal and spatial patterns.

An example of the artifact identification process is illustrated in Figure 9. In this figure, the left panel displays the time courses of 26 independent components extracted from an EEG recording using ICA, while the right panel shows the topographic maps of four selected components (Components 1, 2, 3, and 6) that were identified as artifacts. Component 1 presents a highly focal topographic pattern, restricted to a single electrode location, along with erratic fluctuations in its time course, consistent with impedance artifacts. Component 2 displays high-frequency activity and a spatial distribution in the temporal region, characteristic of electromyographic (EMG) artifacts, usually related to jaw or facial muscular activity. Components 3 and 6 represent strong frontal activation patterns and slow drifts in the time course, which are indicative of ocular artifacts. While the former shows a symmetric frontal topography and a stereotyped, large-amplitude transient in the time course, characteristic of an eye blink, the latter, in contrast, presents a left–right bipolar topographic distribution and a more gradual temporal pattern, which is typical of horizontal eye movements. Because ICA decomposes the EEG signal into statistically independent temporal sources, the distinct time courses of eye blinks and horizontal eye movements allow them to be separated into different components, despite both originating from ocular activity [53].

These components will be identified through the visual inspection of both their temporal dynamics and topographic maps, guided by established criteria from the previous literature on artifact detection using ICA [52,53]. All EEG recordings in this study will undergo a similar inspection procedure, and any components exhibiting spatiotemporal features consistent with those illustrated in Figure 9 will be systematically removed.

**Figure 10:**
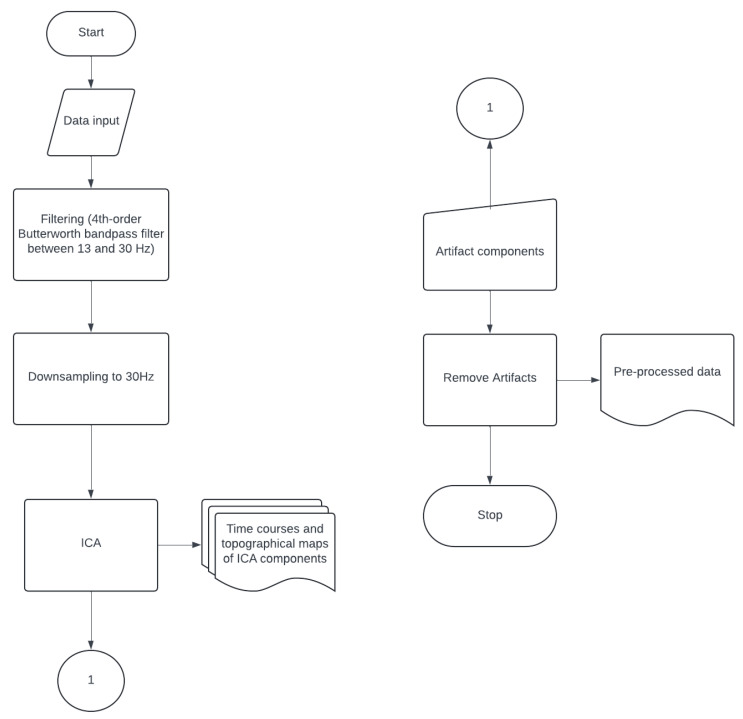
Flowchart of the EEG and EMG data preprocessing pipeline. The raw EEG and EMG signals undergo an initial input followed by bandpass filtering using a 4th-order Butterworth filter (13–30 Hz) and are then downsampled to 60 Hz. ICA is subsequently applied to EEG signals to extract time courses and topographical maps of the ICA components. Artifact components are identified and removed, resulting in the final preprocessed EEG data for subsequent analyses.

This preprocessing workflow is depicted in the flowchart of Figure 10.

**Figure 11:**
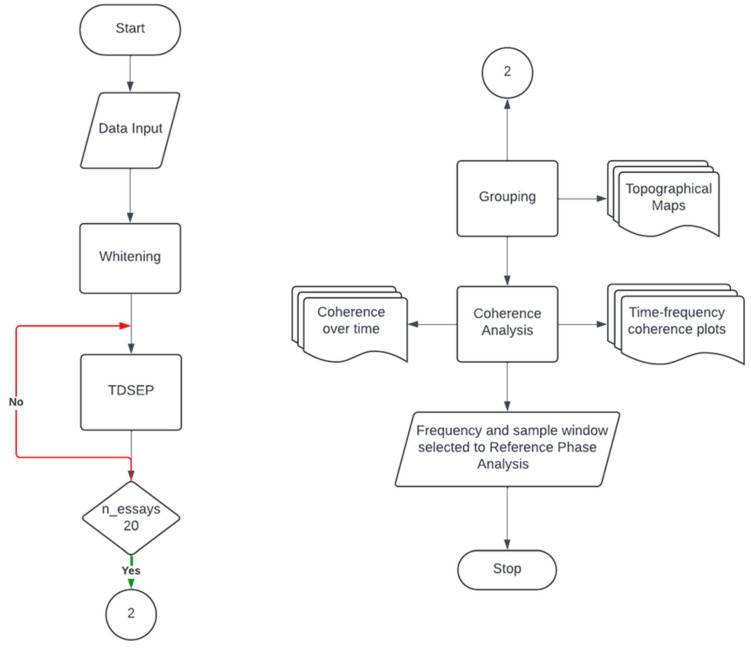
Flowchart of the EEG coherence analysis pipeline. After data input, whitening is applied to improve the computational efficiency of the subsequent source separation. After whitening, Temporal Decorrelation Source Separation (TDSEP) is repeated 20 times (n_essays ≥ 20). The temporally decorrelated sources obtained are grouped using a cross-correlation criterion of 0.9 and coherence analysis between source signals and EMG data is conducted. The outputs of this step include coherence over time, topographical maps, and time–frequency coherence plots. These outputs inform the selection of a specific frequency band and time window, which are used for subsequent Reference Phase Analysis (RPA).

Since the proposed synchronous source separation algorithm focuses on frequency-specific synchronization, it is essential to define the sample window and frequency of interest beforehand. To facilitate this selection for subsequent RPA, we will employ TDSEP, as proposed by Ziehe and Müller [54], to examine the coherence relationships between the EEG sources and the EMG signal. The TDSEP algorithm workflow is illustrated in Figure 11.

TDSEP is chosen for this analysis because temporal decorrelation algorithms provide an effective approach to solving blind source separation (BSS) problems, particularly when the intrinsic temporal structure of the signals is relevant. In contrast, other BSS algorithms that assume statistical independence between sources—such as ICA—may distort physiological coherence and phase relationships in electrophysiological data, making them less suitable for synchronization studies.

The major novelty of the proposed processing pipeline is the application of RPA to investigate corticomuscular coupling at the source level. Traditional CMC approaches and synchronization analyses typically assess corticomuscular coupling between EEG and EMG signals directly at the electrode level, often leading to inaccurate coupling estimates since it is affected by volume conduction. In contrast, RPA applies a source separation step that identifies neuronal sources specifically phase-locked to muscular activity. By computing the EEG source that maximizes the synchrony between the respective EEG source signal and the EMG reference, RPA overcomes the confounding effects present in electrode space and provides a more physiologically accurate analysis of corticomuscular interactions. This method allows for a finer characterization of the neural circuits involved in motor control, offering improved spatial resolution and sensitivity to dynamic, transient synchrony patterns compared to conventional electrode-based coherence and synchrony analyses.

RPA will be applied, as proposed by Almeida et al. [37], to identify a linear transformation of the mixed signals that maximizes phase synchronization with an EMG reference [55]. This synchronization will be quantified using the Phase-Locking Value (PLV) [39], defined as

(2) $$PLV\;=\frac{1}{N}\left|\langle e^{i\Delta \phi \left(t\right)}\rangle \right|$$

where $\langle \cdot \rangle$ denotes the time average operator, $\Delta \phi \left(t\right)$ represents the phase difference between the realizations of the EEG source and EMG signals $s_{1}$ and $s_{2}$, respectively, expressed as $\Delta \phi \left(t\right)=\phi_{1}\left(t\right)-\phi_{2}\left(t\right)$, and N is the total number of time points.

Given the nonstationary nature of EEG and EMG signals, as well as the transient characteristics of synchrony, we will implement a sliding window approach to enhance the reliability of the PLV estimate. The RPA method will employ an adaptive step-size gradient ascent algorithm to maximize the PLV between EEG sources and EMG signals. However, this optimization process is sensitive to initial conditions and the presence of local maxima. To mitigate these effects, RPA will perform 50 iterations per window, each initialized with randomly generated starting points. Consequently, RPA will compute 50 synchronous sources per window, which will then be grouped using a pairwise correlation-based similarity criterion. For intra-window grouping, an absolute correlation threshold of 0.85 will be applied, followed by an inter-window grouping step using a more relaxed absolute correlation threshold of 0.70.

This final grouping procedure will allow us to assess whether the identified synchronous sources are consistent across the entire signal. Additionally, it will help identify the most frequently occurring source, designating it as the most likely candidate for the source of interest.

**Figure 12:**
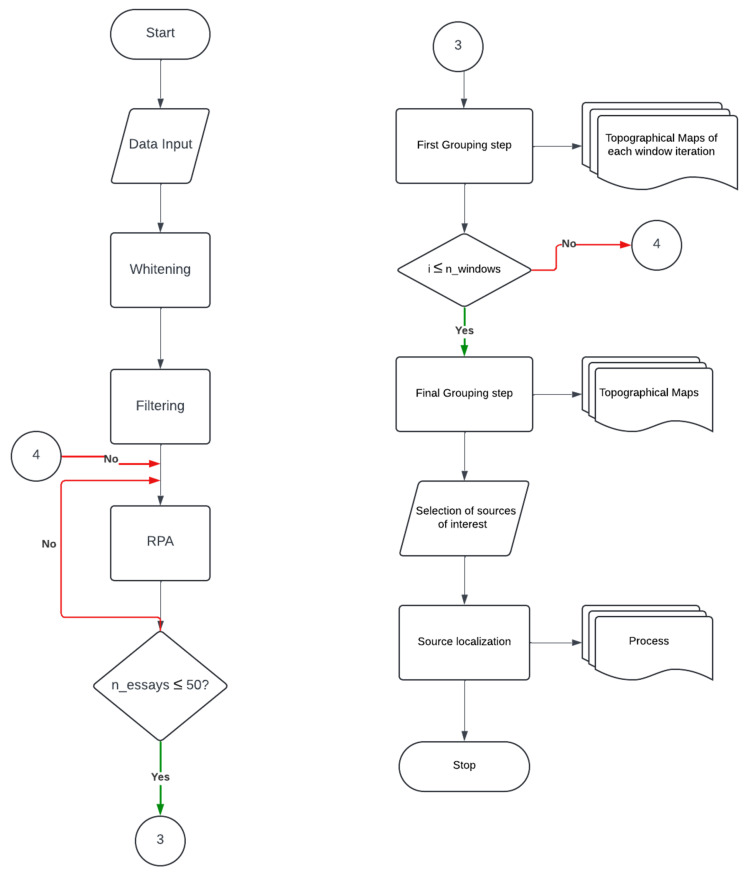
Flowchart of the RPA pipeline. The process begins with data input, whitening, and filtering with a 4th-order Butterworth filter of a 2 Hz range centered on the frequency of interest selected in the coherence analysis. A windowing technique is applied, followed by RPA, which will run repeatedly 50 times with different initial conditions for each window (n_essays ≤ 50). The topographical maps of the 50 RPA sources obtained are grouped. Iterations continue until all time windows (i ≤ n_windows) are processed. A final grouping step is then performed to consolidate the results across windows, producing the final topographical maps used for selecting sources of interest. These selected sources are then localized using the FieldTrip toolbox.

Finally, the FieldTrip toolbox will be employed to localize brain sources exhibiting a consistent pattern of synchrony with muscular activity. The flowchart of the RPA algorithm is presented in Figure 12.

## 3. Expected Results

The proposed methodology is expected to provide valuable insights into brain–muscle connectivity through phase synchrony. The protocol outlined in this study should effectively identify and separate the brain sources exhibiting phase synchrony with muscular activity.

**Figure 13:**
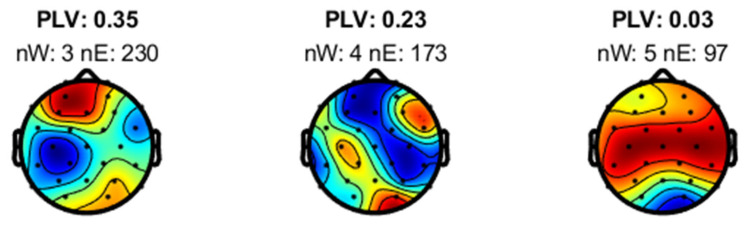
EEG sources separated by RPA using the EMG signal as reference.

Preliminary data from an 11-year-old right-handed healthy participant performing ‘Task 1′ with his right hand showed promising results. Using the EMG signal as a reference, the RPA algorithm identified distinct EEG sources, as illustrated in Figure 13.

**Figure 14:**
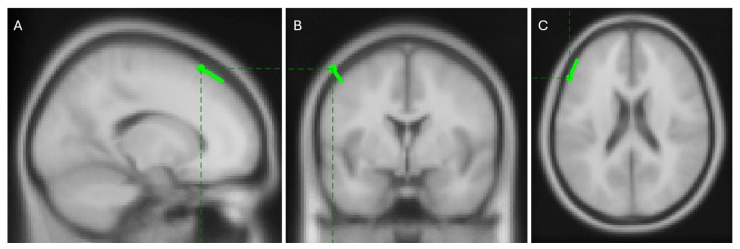
Sagittal (**A**), coronal (**B**), and transversal (**C**) views of the localization of the most synchronous source situated in the left caudal middle frontal area. Images present standard magnetic resonance slices which are not fixed at the dipole position.

Among these sources, the one with the highest PLV, shown as the left-most map in Figure 13, is expected to represent a clear dipolar source located in the brain cortex contralateral to the movement. Localization with the FieldTrip toolbox places this source in the left caudal middle frontal area, as shown in Figure 14. This region is well-documented for its role in manual control [56,57,58,59,60] and its contralateral location aligns with the known physiology of human motor function [61]. These results support the hypothesis that phase synchrony is a strong candidate for explaining corticomuscular control.

It is expected that the proposed methodology, particularly the use of RPA, will enable the detection of physiologically meaningful corticomuscular coupling localized in cortical regions implicated in motor preparation and execution, such as the primary motor cortex and the supplementary motor area [62,63,64,65,66].

Task-dependent variations in EEG-EMG phase synchrony are also anticipated. Tasks involving steady force production, abrupt force changes, and gradual transitions are expected to elicit distinct synchrony patterns, providing a comprehensive characterization of corticomuscular dynamics across diverse motor demands.

Differences in corticomuscular coupling are expected between typically developing children and children with cerebral palsy. These differences are hypothesized to be reflected both in the magnitude of phase synchronization and in the spatial distribution of neural sources associated with motor control.

Preliminary results presented in the manuscript support these expectations, demonstrating the pipeline’s capability to identify phase-locked activity between EEG and EMG signals in cortical areas aligned with motor function, in accordance with the existing literature. Although the comprehensive analysis of a larger dataset lies beyond the scope of this study protocol, the application of the methodology to the control group has yielded consistent outcomes when compared to the preliminary results discussed before, further reinforcing the validity and robustness of the proposed framework.

This protocol, aimed at exploring corticomuscular control by investigating phase coupling between neuronal and muscular activity, will provide crucial insights into the neural mechanisms underlying motor control. Moreover, characterizing corticomuscular synchrony in both time and coupling strength, alongside precise localization of the involved neuronal sources, will be essential to identify pathological corticomuscular coupling patterns. These may serve as biomarkers for motor disorders. Such insights are critical not only for advancing our understanding of motor control but also for developing targeted interventions for children with cerebral palsy, thereby improving patient outcomes and therapeutic strategies.

## 4. Discussion

The preliminary results discussed in the previous section demonstrated promising outcomes for our study protocol. Using the EMG signal as a reference, the RPA algorithm successfully identified distinct EEG sources exhibiting phase synchrony with muscular activity. Among these, the source with the highest PLV was located in the left caudal middle frontal area, a well-known cortical region associated with manual motor control [56,57,58,59,60]. The contralateral activation aligns with known motor physiology, suggesting that phase synchrony is a reliable metric for assessing corticomuscular connectivity. Furthermore, the results indicate that RPA can effectively identify and characterize neural sources that drive motor control, enhancing our understanding of underlying neural dynamics. These findings suggest that the methodology could provide precise insights into motor control mechanisms and potentially identify biomarkers for motor disorders.

These results highlight the potential of our approach to advance beyond traditional corticomuscular connectivity assessments. Unlike conventional studies relying primarily on standard CMC metrics, our method combines phase synchrony analysis with neuronal source separation. While CMC metrics assess oscillatory coupling, their dependence on signal amplitude can inflate connectivity estimates, risking the misinterpretation of neural interactions [39,40]. Standard coherence analyses, typically performed at the sensor level, lack spatial resolution and are vulnerable to volume conduction, which can also inflate connectivity estimates [41,42,43]. Although source localization techniques have been explored to mitigate these effects [22,23,24,25], their application remains limited.

In contrast, our approach uses phase synchrony analysis, inherently independent of amplitude variations, enabling more robust detection of the physiological corticomuscular interactions [35,36]. This is especially advantageous in populations with motor impairments, where conventional coherence metrics may miss subtle but clinically relevant synchrony patterns. Additionally, neuronal source separation based on phase synchrony enhances spatial specificity, effectively reducing spurious synchronization from volume conduction or linear mixing. This refinement allows for the clearer identification of cortical drivers underlying motor activity.

Notably, this study introduces serious gaming as an innovative paradigm to engage participants in controlled, repeatable fine hand motor tasks. This setup was developed to standardize motor task execution and optimally drive corticomuscular interaction, enabling more accurate assessments of motor control in both healthy individuals and children with cerebral palsy.

Consequently, our approach offers a more physiologically grounded evaluation of corticomuscular coupling, addressing traditional CMC limitations and improving the accuracy of motor control assessments in typical and clinical populations.

### 4.1. Novelty of This Study Protocol

This protocol introduces a novel methodological framework that advances the study of corticomuscular control, especially in pediatric populations affected by cerebral palsy.

A key innovation is the use of phase synchrony metrics combined with neuronal source separation. Traditional CMC and phase synchronization analyses, though insightful, present critical limitations, including a dependence on signal amplitude and vulnerability to volume conduction. These constraints can obscure true neural connectivity, leading to potentially misleading interpretations of corticomuscular interactions. Unlike conventional CMC, phase synchrony assessed via RPA detects transient, frequency-specific coupling between cortical and muscular signals independent of amplitude.

The experimental paradigm is also novel, integrating diverse motor tasks within a serious gaming framework designed to analyse different motor control aspects. Real-time visual feedback from the gaming interface engages participants in controlled, repeatable tasks, reducing inter-subject variability. Some tasks use both dominant and non-dominant hands to explore hemispheric asymmetries. To isolate neural-driven synchronization from cognitive and visual influences, certain tasks are performed without the gaming interface, avoiding the confounding effects of visual feedback.

Focusing on pediatric populations, specifically children with cerebral palsy, addresses a critical gap in research, since corticomuscular studies typically target adults, leaving developmental aspects underexplored. By targeting fine motor skills via an engaging gaming platform, this protocol aims to elucidate the neural mechanisms behind motor impairments in cerebral palsy, offering valuable insights into typical and atypical motor development.

In summary, the protocol integrates advanced phase synchrony analysis, neuronal source separation, and a motor task set tailored for corticomuscular studies. These methodological advances overcome the core limitations of traditional CMC analyses, enabling more precise, meaningful interpretations of corticomuscular connectivity in children with cerebral palsy. This approach enhances assessment reliability and provides a robust protocol to study the neural bases of motor dysfunction, potentially informing future therapies.

### 4.2. Clinical Implications

The findings of this study hold substantial clinical relevance for the rehabilitation and treatment of children with cerebral palsy. By examining the patterns of phase synchrony between cortical and muscular activity, this work contributes to a deeper understanding of corticomuscular connectivity impairments associated with motor dysfunction in this population. Such insights may enable the identification of specific neurophysiological deficits underlying abnormal motor control.

This knowledge provides a foundation for the development of targeted neurorehabilitation strategies, including neurofeedback protocols, brain–computer interface (BCI) applications, and optimized physiotherapeutic interventions that are tailored to the individual’s neural profile. Enhancing motor control through the modulation of brain–muscle communication could lead to more effective and personalized interventions.

Furthermore, the identification of cortical sources exhibiting phase synchrony with muscle activity may serve as objective biomarkers for assessing the severity of motor impairment and monitoring the efficacy of therapeutic interventions over time. Comparing these neural patterns with those of typically developing peers offers a normative framework for clinical benchmarking.

Ultimately, this study contributes to the advancement of evidence-based, precision rehabilitation approaches, supporting clinical decision-making and improving functional outcomes and quality of life in children living with cerebral palsy.

### 4.3. Limitations

Despite its potential contributions, this study protocol has certain limitations that may affect the generalizability and interpretability of its findings. First, the analysis is restricted to hand motor tasks. While informative, this narrow focus may not fully capture the broader spectrum of motor deficits observed in children with cerebral palsy, particularly those involving lower limbs or complex movements. Furthermore, the protocol includes only tasks that participants with cerebral palsy can execute, which introduces a selection bias toward individuals with relatively preserved motor function and corticomotor connectivity.

The inability to assess motor tasks that participants cannot execute—often the most impaired or clinically significant—limits the protocol’s ability to characterize the full range of motor dysfunction in cerebral palsy. This constraint reduces the protocol’s applicability to individuals with more profound impairments and may obscure important neural mechanisms underlying more severe motor deficits.

In addition, the protocol’s focus on frequency-specific simplifications may fail to capture critical aspects of neural dynamics, as emerging research has emphasized the importance of cross-frequency phase synchronization in corticomuscular control [67,68,69]. Neglecting these interactions could lead to an incomplete understanding of how different frequency bands interact to support motor function, particularly in pathological conditions like cerebral palsy.

### 4.4. Future Directions

Future research should expand the assessment of corticomuscular connectivity to more motor tasks and clinical populations. While this study focuses on hand tasks in children with CP, extending to lower limb movements and complex bilateral activities would provide a comprehensive evaluation. Investigating other neurological conditions with motor dysfunction, such as Parkinson’s disease and stroke, could reveal disease-specific connectivity disruptions and biomarkers, supporting targeted rehabilitation.

Additionally, future studies could enhance the RPA algorithm by integrating cross-frequency phase coupling metrics. Such an analysis would potentially detect interactions across frequency bands (e.g., theta–gamma, alpha–beta), revealing the complex neural communication patterns underlying motor control. Such an approach could deepen our understanding of how neural oscillations coordinate during motor execution and adaptation, especially in clinical populations with abnormal motor control.

## Data Availability

The serious gaming interface code is available in https://doi.org/10.5281/zenodo.11245416.
